# Fetal fibular hemimelia with focal femoral deficiency: A case report

**DOI:** 10.4274/tjod.galenos.2019.89990

**Published:** 2019-10-10

**Authors:** Betül Yakıştıran, Orhan Altınboğa, Tuncay Yüce, Ali Turhan Çağlar

**Affiliations:** 1 University of Health Science, Ankara Zekai Tahir Burak Women’s Health Practise and Research Hospital, Clinic of Obstetrics and Gynecology, Ankara, Turkey

**Keywords:** Aplasia, fibula, fetal development, abnormalities

## Abstract

Fibular hemimelia (FH) is a congenital deficiency in which a part or all of the fibular bone is hypoplastic or aplastic and associated with hypoplastic tibia and foot anomalies. The main differential diagnoses include proximal focal femoral dysplasia, Femur-Fibula-Ulna syndrome, and Femoral Hypoplasia-Unusual Facies syndrome. Proximal focal femoral dysplasia, which has a short, angulated femur with normal mineralization may be associated with FH. We report a case of unilateral FH with focal femoral deficiency detected at 18 weeks of gestation during a routine ultrasonographic anatomic screening. Sonographic findings were a unilateral short femur (1.8 cm, 3 weeks shorter than expected for gestational weeks), agenesis of ipsilateral fibula and angulation of ipsilateral tibial shaft. During a routine ultrasonographic anatomic scan, all the long bones are carefully measured and evaluated. Long bone shortness can be a part of syndrome or an isolated finding.

## Introduction

Fibular hemimelia (FH) is a congenital deficiency in which a part or all of the fibular bone is hypoplastic or aplastic and associated with hypoplastic tibia and foot anomalies^(1)^. FH is one of the most common congenital deficiencies of the long bones with an estimated incidence between 5.7-20:1.000.000^(2)^. FH is most often sporadic and may be part of more complex syndromes. There are numerous classification systems for FH; Achtermann and Kalamchi, Coventry and Johnson, and Stanitski. The Achtermann and Kalamchi classification is more commonly used and this classification is based on the degree of fibular deficiency present^(3)^. In Type 1, the fibula is present but hypoplastic, whereas in Type 2, it is completely absent. Type 1 is divided into 1A, where the proximal fibular epiphysis is distal to the tibial growth plate, and the distal fibular growth plate is proximal to the talar dome, and 1B, where there is a partial absence of the fibula and there is no distal support for the ankle joint. In Type 2 deformities, bowing of the tibia is more severe than in Type 1^(3)^. The main differential diagnoses include proximal focal femoral dysplasia, Femur-Fibula-Ulna syndrome, and Femoral Hypoplasia-Unusual Facies syndrome. Proximal focal femoral dysplasia, which is with short, angulated femur with normal mineralization may be associated with FH^(4)^.

We report a case of unilateral FH detected at 18 weeks of gestation during a routine ultrasonographic anatomic screening.

## Case Report

**Figure 1:**
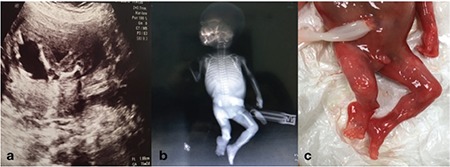
The right leg. a) Sonography of the right leg, showing the shortening of the right femur and abnormally angulated tibia, b) X-ray of the lower limb shows proximal focal femoral deficiency, short and angulated tibia and absence of fibula, c) post-abortus material visualization confirming the abnormalities of the right leg and right foot

A gravida 2, para 1-0-0-1, 28-year-old woman was referred to our high-risk obstetric clinic at 18 weeks 2 days of pregnancy. Detailed two-dimensional (2D) ultrasound examination was performed with a 2-7 mHz abdominal ultrasound transducer (Voluson™ 730 Pro; GE Healthcare, USA). Sonographic findings were a unilateral short femur (1.8 cm, 3 weeks shorter than expected for gestational age), agenesis of ipsilateral fibula, and angulation of ipsilateral tibial shaft (Figure 1a). The measurements of contralateral tibia, fibula, femur, and the length of the upper limbs were within the normal range according to the gestational age. No other facial morphology, cardiac, neurologic, gastrointestinal, and genitourinary system abnormalities were identified. There was no maternal history of diabetes, drug exposure, viral exposure, and trauma during this pregnancy. The patient elected for pregnancy termination. Abortion was conducted with misoprostol and a 380 g male fetus was aborted and the intact abortus material underwent pathologic and genetic examination. Post-abortal skin biopsy results indicated normal karyotype. Post-abortal X-rays of the fetus confirmed the sonographic findings (Figure 1b, 1c).

## Discussion

FH is defined as shortening or absence of fibula and is a rare longitudinal deficiency. It ranges from mild deficiency to complete absence of fibula. FH can also coexist with Fetus-Fibula-Ulna syndrome, intercalary hemimelia of the fibula, congenital deficiency of proximal femoral focal deficiency, and congenital short tibia with absent or dysplastic fibula. The main sonographic findings are deformed or absent fibula with normal mineralization, ossification, shortened or anteriorly curved tibia, significant shortening of the femur, and foot anomalies^(2,5)^. The unilateral form is approximately 60-80% of all cases and the right side is more commonly affected than the left^(6)^. Embryologic development and documentation by sonography of upper and lower limbs takes place nearly at the end of the eighth to tenth weeks of the pregnancy. Interconnection of complex several regulatory proteins such as bone morphogenic proteins, fibroblast growth factor, hedgehog proteins, and homeobox factors are prerequisites for limb development^(7)^. The definite etiology is unknown, but the proposed theory is disruption of vascular development resulting from the interruption of blood flow and muscular development^(8)^. When a congenital limb deficiency is diagnosed, the fetus should have a thorough anatomic scanning for other system anomalies. Longitudinal limb deficiencies can occur in isolation but sometimes may be part of a syndrome. The parents should be asked and examined for limb anomalies in order to clarify any familial transmission. A detailed pregnancy history including medications, viral exposure, drug use, trauma, diabetes mellitus, and chorion villus sampling in the early weeks of pregnancy may be helpful in identifying etiologic factors. For differential diagnosis of FH, fetal anatomic scanning can be performed by 2D and 3D ultrasonography^(7,8)^. When a long-bone shortness is determined, all fetal long bones should be measured. Also evaluations must be performed for the fetal face profile, cardiovascular system, neurologic, genitourinary, and gastrointestinal system to determine any co-existing syndromes. After presumptive diagnosis, early evaluation through a multidisciplinary approach with a geneticist and a pediatric orthopedic surgical team is an important component in making a management plan. Various classification systems have been made for FH and these classification systems can aid parental counseling and surgical procedure decisions. Although the Achtermann and Kalamchi classification system is more commonly used in the postnatal period,^(2,3)^ there are mainly three types of absence of fibula: Type 1 (10% of all cases) is characterized by total or partial absence of the fibula and mild or no bowed tibia. Type 2 (35% of all cases) is characterized by unilateral absence of the fibula, anterior bowing of the tibia, and significant shortening of the leg. Type 3 (55% of all cases) includes cases with uni/bilateral absence of the fibula with same-leg and foot deformities^(5,8)^. According to our sonographic findings (absence of the right fibula, bowed tibia, shortening femur, and valgus deformity), our case was classified as Type 3 FH with co-existing proximal focal femoral deficiency. Its association with FH has been reported in approximately 50% of cases. The prognosis of FH depends on the severity of fibular deficiency, associated femoral malformations, and foot, ankle or knee deformities^(2)^. Treatment options include amputation (the preferred management of a child with absence of fibula) and orthostatic or prosthetic support (to maintain limb length equality)^(2,9)^. It is individualized for each case and undertaken in experienced centers with access to a multidisciplinary team including a pediatrician, physical therapists, and orthopedists. Patients with FH are not associated with mental retardation, but both treatment options for children with FH result with a lower quality of life. In the literature, several cases are reported in which the parents elected for termination of pregnancy before fetal viability, as in our case, after parental counselling. Although FH is not a definite termination indication, detailed information about the severity of limb deformity and treatment options can aid parents in decision-making regarding the continuity of pregnancy.
